# Reflection in practice: How can patient experience feedback trigger staff reflection in hospital acute care settings?

**DOI:** 10.1111/hex.13010

**Published:** 2019-12-19

**Authors:** Jennifer Jones, Julian Bion, Celia Brown, Janet Willars, Olivia Brookes, Carolyn Tarrant, the PEARL collaboration

**Affiliations:** ^1^ Health Sciences department University of Leicester Leicester UK; ^2^ Intensive Care Medicine University of Birmingham Birmingham UK; ^3^ Warwick Medical School The University of Warwick Warwick UK; ^4^ Research Development and Innovation University Hospitals Birmingham NHS Foundation Trust Birmingham UK

**Keywords:** acute care, patient experience, staff reflection

## Abstract

**Background:**

Patient and staff experiences provide important insights into care quality, but health systems have difficulty using these data to improve care. Little attention has been paid to understanding how patient experience feedback can act as a prompt to reflection in practice in the clinical setting.

**Objective:**

We aimed to identify the ways in which different types of patient experience feedback act as a trigger or prompt for engagement in reflection in clinical practice in acute hospital settings and identify important considerations for enhancing the value of patient experience data for reflective learning.

**Methods:**

We conducted an ethnographic study in eight acute care units in three NHS hospital trusts in England, including 140 hours of observations and 45 semi‐structured interviews with nursing, medical and managerial staff working in acute medical units and intensive care units. The data were analysed thematically.

**Findings:**

We distinguished between formal patient experience data sources: data purposively collected and collated to capture the patient experience of care, generally at organizational level, including surveys, complaints and comments; and informal sources of feedback on the patient experience recognized by staff alongside the formal data. We also identified patient narratives as an ‘in between’ source of data. The impact of different types of patient feedback in triggering reflection primarily depended on the extent to which the feedback was experienced as personally relevant, meaningful and emotionally salient.

**Discussion:**

Patient experience feedback is multi‐faceted, but our study suggests that all types of feedback could be harnessed more effectively to prompt reflection.

## 1 INTRODUCTION

Patient and staff experiences provide important insights into care quality, but health systems have difficulty using these data to improve care. Evidence suggests that organizations struggle to manage the data they collect and to make improvements based on patient experience feedback, and that clinicians often fail to change their practice based on patient experience feedback^1^, ^2^. One particular challenge in acting on patient experience feedback is that, when patients express dissatisfaction with their care, they often identify problems with staff‐patient interactions.^3^ Around one‐third of patient complaints relate to staff‐patient relationships^4^ such as communication, empathy, courtesy, consideration and compassion demonstrated by front‐line staff; these aspects of care are critical for positive patient experiences.^5^, ^6^, ^7^ Evidence suggests, however, that patient experience data currently available in the NHS tend to be used to stimulate changes in care processes which are technical in nature, rather than tackling the more difficult task of changing clinician behaviour.^8^

Although staff are unlikely to intentionally behave in ways that are detrimental to the patient experience, they may lack insight into how their behaviours affect patients or how to modify those behaviours. One approach for promoting insight and change is reflective learning. Reflection involves engagement in retrospection, self‐evaluation and re‐orientation^9^ based on individuals' own experiences or feedback on their performance, or the experiences of others. Reflection can take different forms. It can be an individual or group activity.^10^ It may happen ‘in action’ when an event gives immediate cause for thought or can be a deliberative process looking back ‘on action’ to generate new perspectives and intentions for change.^11^ The idea that reflection will lead to learning and improvement is based on the work of Dewey from the 1930s^12^ and continued with models such as Schön and Gibbs designed to support reflective practice.^13^, ^14^, ^15^, ^16^ Whether reflection prompts learning and change has been questioned, although some studies have identified changes in behaviour as a direct result of reflection taking place within clinical practice settings.^17^, ^18^, ^19^, ^20^ Reflective practice is now mandated for most health professionals, with documented evidence of reflecting on patient and colleague feedback required for continuing professional development and revalidation. Despite the focus on retrospective written reflection, increasingly, arguments are being made that reflection, and in particular reflection in action, should instead be fully embedded within the multiple contexts of clinical practice.^21^This requires clinicians to make reflection part of everyday routines and practices, and develop skills to recognize and act on prompts or triggers for reflection.^22^

Reflection requires a prompt or trigger: ‘a “disorientating dilemma” or a period of uncertainty in what should be done—that leads to exploration with a critical perspective, challenging underlying assumptions, beliefs, motives and values’.^22^ By definition, reflection involves a switch from automatic processing, to enhanced cognitive awareness and deeper processing and learning.^12^ The ability of a trigger to prompt an emotional response is considered to be critical for stimulating reflection; indeed, reflective learning is argued to involve an interplay between cognition and emotion.^23^

In principle, feedback about patients' experiences can be a powerful trigger or prompt for reflection, opening up the opportunity for personal insight development and changes in attitudes and practice. Several studies have assessed how reflective learning has been enhanced by providing a patient experience trigger and measuring its impact, usually as an intervention study. For example, video vignettes have been used by dental undergraduates,^24^ facilitated patient experience feedback has been shown to improve nursing care,^25^ and studies have shown how patient narratives can serve as reflective devices for health‐care professionals.^26^, ^27^ Qualitative research has identified patient experience feedback as a trigger for reflection in everyday clinical practice, along with other triggers, including difficult interpersonal interactions with patients and their families or between staff members; uncertainty about clinical care; unexpected clinical outcomes; emotional responses to high stakes situation; and external feedback on performance^28^

A wide variety of patient experience data is available in the health‐care setting, ranging from surveys and questionnaires, to compliments, informal feedback to PALS and suggestion boxes,^29^, ^30^, ^31^ It is not clear, however, that current approaches to managing feedback about patients' experiences maximize the value of this feedback as a trigger for reflection in practice.^32^ Little attention has been paid to understanding how different types of patient experience feedback can act as a prompt to reflection in practice in the natural clinical setting, rather than as part of an intervention study.

We aimed to identify the ways in which different types of patient experience feedback act as a trigger or prompt for engagement in reflection in clinical practice in acute hospital settings and identify important considerations for enhancing the value of patient experience data for reflective learning in clinical practice.

## 2 METHODS

### 2.1 Setting

We conducted an ethnographic study of reflection on patient experience feedback in eight acute care units in three NHS hospital trusts in England, including observations and interviews with staff working in acute medical units (AMUs) and intensive care units (ICUs), as part of the Patient Experience and Reflective Learning (PEARL) project.^33^ The three trusts were purposively selected as serving diverse, predominantly urban populations with high‐volume workloads. The eight participating units included three AMUs and five ICUs on four hospital sites. The core project team involved patient and carer representatives as active team members; local project teams also included patient and carer representatives who had experience of care in the participating units (named in the acknowledgements). The PEARL Project received ethics approval from the London Brent Research Ethics Committee (REC Ref 16/LO/224).

### 2.2 Sample

Interview participants were selected to include staff from across the different units and to include nursing, medical and managerial staff with different levels of organizational and individual involvement in patient experience data and reflective practice.

### 2.3 Data collection

Observations and interviews were conducted between May and December 2017, and focused on exploring how patient experience feedback was collected and used, how and why staff reflected on patient feedback, and the structures, processes and activities that facilitated or obstructed staff engagement in reflection in clinical practice. Over 140 hours of observations were conducted in the acute care units by JW, a non‐clinical researcher with extensive qualitative research experience. Observations involved the researcher spending time in the clinical setting, observing day‐to‐day practice, shadowing staff while they performed their tasks, talking to staff informally in clinical and social areas and attending relevant meetings (eg patient coffee mornings and clinical governance meetings). The researcher observed and questioned staff specifically about activities around the collection and use of patient experience data, and engagement in and support for reflection in practice. The researcher documented 81 informal conversations with a wide range of staff about feedback of patient experience data and reflection on patient experience. We collected relevant documents such as newsletters and photographs of patient experience displays within the units. The researcher made written field notes during observations, which were summarized as audio‐recorded debriefs.

Semi‐structured interviews were conducted by JW with a purposive sample of 45 members of staff, between 14 and 16 in each hospital trust. Interviews were conducted in two rounds. Round 1 (36 interviews) focused on the collection and use of patient experience data and reflection on patient experiences. Round 2 (nine interviews) focused in on reflection in practice—triggers, barriers and facilitators—to explore emergent themes around reflection in practice in more depth. Informed consent was obtained for interviews. Interviews were recorded and transcribed verbatim, and anonymized during transcription.

### 2.4 Topic guides

Observations were guided by a sensitizing observation guide, which focused observations on the collection and use of patient feedback, and the structures, process and activities in place in sites that impacted on reflection in practice. The topic guide was used to help anchor the observations to the research questions while leaving the researcher room to pursue lines of enquiry in the field.

The topic guides for interviews explored staff experience of the collection and use of patient experience data, how feedback on patient experience stimulated reflection, and the barriers and facilitators to reflection in clinical practice. The topic guide was modified for the second round of interviews.

### 2.5 Analysis

We took a thematic analysis approach to analysing the data.^34^ Interview and observational data were analysed together through the analysis process. A subset of interviews and observation debriefs were read in close detail by JJ and CT and then open‐coded to create a coding frame and initial thematic categories; these were discussed with the wider study team. The coding frame was then applied to the remaining interviews and observational data transcripts. The coding frame was modified and extended as new themes arose. NVivo 11 software was used to support the management, coding and querying of the data. We used narrative summaries and visual displays to interpret and synthesize the data.

We conducted regular team debriefs during the data collection and analysis period (involving JJ, JW, and CT) to reflect on emerging findings and guide ongoing data collection and discussed findings with the wider team. As thematic analysis showed similar staff responses regardless of site or setting, we did not do a comparative analysis between hospitals or between ICUs and AMUs. Differences in the types of feedback available to staff in ICU and AMU settings are discussed as part of our findings.

## 3 FINDINGS

We distinguish between formal patient experience data sources: data purposively collected and collated to capture the patient experience of care (generally at organizational level, including surveys, complaints and comments); and informal sources of feedback on the patient experience recognized by staff alongside the formal data. We also identified patient narratives as an ‘in between’ source of data. These three sources of patient experience feedback differ in their intrinsic qualities and hence their utility for triggering reflection and the extent to which they can be systematized as part of strategies to promote reflection in practice.

### 3.1 Formal patient experience feedback

Formal sources of patient experience feedback, generated through organizational activities including patient experience surveys and systems for recording complaints, were shared widely with front‐line staff through poster displays, reports, emails and information in meetings. Formal patient experience feedback was seen by staff as having value for organizational performance monitoring and identifying areas for quality improvement, but tended to be less impactful in stimulating individual reflection and attitude change in practice. This was particularly the case for surveys employing quantitative or semi‐quantitative methods without qualitative or narrative components.

#### 3.1.1 Lack of meaningfulness or emotional response to survey data

Staff identified issues that limited the extent to which they were motivated to engage effort in processing feedback from patient experience surveys including concerns about local or personal relevance, timeliness and lack of granularity in the data. In the main, however, survey data feedback that was purely numerical and lacked free‐text components was relatively ineffective for promoting reflection and individual attitude and behaviour change, because the personal meaningfulness was limited, and affective cues generating an emotional response were lacking.

I had a conversation with an HCA […] And she went "oh, I think we display [patient experience survey feedback]", and then she went over to the board,"this is it" And […] she was looking at it then and she was saying "well but that doesn"t mean anything to me" (observation)

We have figures about [patient experience surveys] and I look at them and I just think I'm not acting upon that, I'm not changing my practice based upon that (interviewee 009, nurse)

By contrast, qualitative feedback such as survey free‐text or individual complaints or compliments triggered spontaneous individual reflection and prompted changes in individuals' attitudes and practice. Formal feedback prompted reflection when staff members were able to relate to it personally and experienced an emotional response that led them to think carefully about their actions and future practice in their interactions with patients.

The forms I've read with the patient experience, I've noticed that sometimes they [feel] like, that they're treated sometimes by their illness rather than as a person. […] That's made me feel awful that person's felt like that. So on reflection, I think it's made me try and personalise care, and try and remember at the end of the day there's a person in that bed, and we're not just treating what they've come to hospital with. (interviewee 032, nurse)

#### 3.1.2 Reflecting on formal feedback: Feedback needs to be made meaningful and relevant

Staff suggested that formal feedback could be used purposefully to stimulate reflection, but needed to be curated and digested to make it meaningful and relevant to staff. Also, efforts were required to engage staff in reflecting on formal sources of patient experience such as survey feedback or complaints as part of routine clinical practice, including allocating time for processing and reflecting together on the information. Having organizational systems in place to actively disseminate feedback and encourage reflection made it more likely that formal patient experience feedback would be recognized as a prompt for reflection, and that opportunities for reflecting based on this feedback would be taken up.

We produce a monthly complaints mailer […] essentially saying these are two or three themes that we've identified through complaints, this is what's happened […] reflect on it, reflect on the practice in your area, could this happen essentially to your patients? (interviewee 047, admin)

We observed, however, that on the whole organizational efforts gravitated towards highlighting and acting to address *negative* feedback and identifying areas for improvement. Staff described how their organizations disseminated negative feedback from formal systems, particularly complaints, to promote cross‐organizational learning and improvement, but that this same approach was not always taken to ensure positive feedback was shared across the organization. We also observed examples where potential triggers for reflection and learning based on positive feedback were passed over.

At the clinical governance meeting […] they'd just spent an hour discussing incidents […] but when it came to the compliments literally it was really skipped over. [Feedback from the patient was read out:] "[Person 1] 's kind words and use of hands to squeeze was gratefully appreciated." And the staff at the meeting went ‘oh, great to squeeze hands’. And they sort of dismissed it really. (observation)

### 3.2 Informal feedback on the patient experience

Alongside formal patient experience feedback solicited by the organization, staff recognized a large and diverse field of informal sources of feedback. Usually unsolicited, this included conversations with patients and relatives at the bedside, thank you cards and gifts, a hug from a patient or relative. This type of feedback was more often described as personally relevant and highly emotionally engaging, and as a valuable trigger for stimulating spontaneous reflection.

#### 3.2.1 Informal feedback from patients and colleagues had relevance and emotional salience

This informal feedback received by staff from patients and relatives as part of their daily practice was usually valued and had the potential to incur a sense of personal responsibility in staff to consider their behaviours and relationships with patients. Staff described the discomfort of receiving personal negative feedback; this could motivate them to reflect and elaborate on the experience and think about the implications for their practice.

I said to the nurse [about a patient] "I think he's definitely got diabetes ‘cause he's got a large BMI" and then later on the patient said "Oh I heard you saying large BMI" and told me how he found it quite offensive and how he was upset by me saying that. […] So I think that experience, has changed the way I talk about patients (interviewee 056, doctor)

Staff also recognized that their colleagues could provide insight into the way they communicated and engaged with patients and how this impacted on the patient experience. Although staff may not always be comfortable in speaking out to colleagues about their practice, feedback from colleagues could be a valuable stimulus for reflection on and improvement in relational aspects of care.

The nurse said to me that "the family said that you were not believing them." […] I thought, because I was in stress probably I asked a question more than two or three times. So […] from then on I take my time when I interact with them. So, I do reflect. And that, that has obviously […] changed my approach. (interviewee 026, doctor)

Staff recognized that staff groups had different opportunities for informal feedback: nurses felt that they were more likely to get informal feedback from patients and relatives at the bedside, positive feedback in particular, whereas doctors felt they often missed out on this opportunity. Informal feedback may not even reach staff, meaning they have no opportunity for the reflective learning that could be triggered.

And this one particular doctor said to me "even if a patient may have made a comment to a nurse about ‘oh, wasn't the doctor lovely’, that won't get fed back to the doctor, because it's not [nurses'] priority to do that, and the nurses are too busy. […] That feedback just doesn't reach them". (observation)

Staff in AMU felt they were less likely than those working in ICU to have the opportunity to build rapport with patients and their relatives due to the short length of stay and felt that they were less likely to get this type of informal feedback from relatives or patients under their care.

#### 3.2.2 Power of positive feedback

Staff described the powerful impact of informal positive feedback for reflection and learning. Informal positive feedback on patient experience, whether in the shape of a comment from a patient or colleague, a thank you card, a box of chocolates or a hug from a relative, often did more than just make staff feel good. Such feedback could have an impact by stimulating staff to reflect on what they had done well and generate learning about aspects of their practice they should maintain and develop. Staff in ICU described how positive feedback helped assuage their fears about whether they were ‘doing the right thing’ and to reinforce for them the value of the sometimes distressing treatments and interventions they had to implement.

You'll get a card or a letter, maybe months down the line that […] they've appreciated the care that the patient's received and the time we've given them, the discussions that we've had, how open we've been. And having that at least takes some of the sting out of the […] moral distress […] that you feel ‐ that you're torturing [patients in ICU], with the best of intention, but you're torturing in what you do. (interviewee 011, nurse)

Staff accounts demonstrated how positive feedback could be a powerful source of learning in terms of bringing their attention to what they were doing well and reinforcing aspects of their practice that contributed to positive patient experiences. Positive feedback also contributed to staff well‐being and a sense of worth in their professional role.

#### 3.2.3 Reflecting on informal feedback: Recognizing and responding to a trigger

Reflection on informal feedback could be unstructured: staff commonly described thinking through a trigger (such as bedside feedback from a patient or colleague) themselves or discussing with colleagues, and in itself this could generate valuable learning and impact on practice in their future interactions with patients. Staff sometimes also used informal triggers as the basis of more formal reflective activity, often linked with the requirement for them to demonstrate reflective learning as part of revalidation or continuing professional development.

Although informal feedback was seen as highly powerful, it is serendipitous: opportunistic and unsystematic. Precisely because of the informal and unsystematic nature of this feedback, the use of it for reflection was dependent on staff being able to recognize it as a prompt or a trigger for reflection, to manage their own emotional reactions to the feedback (which could include defensiveness and denial in the case of negative feedback) and to have the mental capacity and ability to engage in reflection either in the moment or at a later point in time, which could be difficult when staff were tired or stressed.

It's the ability of the individual to accept that and I suppose if I heard anything negative or bad, your initial reaction is "they're wrong"! (interviewee 005, admin)

### 3.3 Patient narratives—‘in between’ feedback

Patient narratives were identified by staff as impactful for stimulating reflection; this source of feedback sat between the formal patient experience data ‘economy’ and the milieu of informal sources of feedback that staff were exposed to in their day‐to‐day practice. Staff described initiatives that elicited patient experience of care directly from the patients themselves in the form of stories or narratives. In some cases, these initiatives involved purposively identifying and using narratives as a prompt for learning, and in others, the reflection and learning were incidental. An example of the former was the collation and use of video narratives from patients about their experiences, to trigger reflection and learning. Incidental reflection arose in the case of patient coffee mornings, observed in one of the participating ICUs. These coffee mornings were arranged for patients who had stayed in ICU to return and talk about their experiences; the primary purpose was to support the patient's rehabilitation through helping them to reconstruct what had happened to them while in the hospital. An unintended consequence was that staff got to hear first‐hand about the patient experience in the ICU. Staff gained considerable insight from hearing patients' personal stories and found that they were challenged to think more deeply about their attitudes and behaviours, and as a result had changed their approach to communicating and interacting with patients in the ICU. These types of activities, where patients return to the ward to recount their experiences, did not happen on AMUs.

I feel like I've certainly become more empathetic towards patients […] after [coffee morning] and reading the experiences online. I actually, I feel like I take it more seriously, […] if there was anything that we can do to help them sleep better, because obviously sleep deprivation can increase the chance of hallucinations. I also find that I do regularly orientate my patients more now than I ever have. (interviewee 012, nurse)

## 4 DISCUSSION

In this study, we used interviews and observations in acute care settings to assess how staff used feedback from patients to reflect, learn and modify their behaviour. We categorized patient experience feedback into two broad categories: formal feedback and informal feedback. Formal feedback which was collected and collated at organizational level (eg through patient surveys) had limited value for triggering reflection unless efforts were made to make it meaningful and flag it as a stimulus for reflection, and opportunities created for staff to take time to reflect on the feedback. Informal feedback (such as bedside comments and gifts of thanks—sometimes considered as ‘soft’ data^35^) was more likely to trigger spontaneous reflection but access to this type of feedback and use of it for reflection in practice was highly unsystematic. In between these two categories were patient stories—actively solicited and sometimes (but not always) purposefully used to stimulate reflection and learning.

The impact of different types of patient feedback in triggering reflection primarily depended on the extent to which the feedback was experienced as personally relevant, meaningful and emotionally salient.^23^ This finding is in line with theory‐based predictions about the influence of different types of message in changing attitudes and behaviour, in particular, that messages perceived as personally relevant are more likely to prompt deeper processing.^36^ We also identified the value of positive feedback for reflection and learning. When we observed discussion of formal patient feedback, there was a strong tendency to focus on the negative, with efforts to try to identify concrete lessons for improvement and change. Positive feedback attained through organizational patient feedback systems, while acknowledged, was commonly overlooked in terms of its potential for generating learning—perhaps because it did not highlight things that needed ‘fixing’, in line with quality improvement goals. In contrast, staff described many examples of positive informal feedback, and how this had supported their learning, reinforced their practice and provided reassurance about their approach to care. We also identified that access to the types of feedback that are most impactful in stimulating reflection could vary between staff groups and settings. In particular, staff working in ICU settings described having more access than AMU staff to informal and individual patient feedback, such as through bedside comments and coffee mornings, providing them with more potential triggers for reflection.

Patient experience feedback is multi‐faceted, but our study suggests that all types of feedback could be harnessed more effectively to prompt reflection. This could include active efforts to maximize the value of formal feedback as a trigger for reflection, through work to make it meaningful and emotionally salient. Ensuring the feedback is comprehensible, the local relevance is made clear, and individual patient experiences provided verbatim alongside graphs and percentages, is likely to enhance the value of formal feedback for reflection, not just for quality improvement. Our findings also highlight the importance of focusing on sharing and learning from positive feedback, to reinforce or enhance current practice. In addition, expanding opportunities for staff to hear patient stories, capitalizing on serendipitous feedback and engaging in efforts to purposefully share informal feedback to enable collective learning, will help increase the exposure of staff to effective triggers for reflection and learning. Key study findings are included in Box 1.

BOX 1 1 **Key study findings**

- Patient experience feedback has most value for stimulating reflection if it is personally relevant, meaningful, and emotionally salient.
- Positive feedback has value for reflection and reinforcement of good practice, as well as providing comfort and reassurance to staff.
- Informal or serendipitous feedback can be a powerful trigger for reflection but may be overlooked in terms of its potential for generalisable learning
- Organisations should consider ways to maximise the capabilities and opportunities for staff to use feedback, particularly informal and serendipitous feedback, for reflection and improvement.

This paper has focused on how staff respond to different types of patient feedback as potential prompts or triggers for reflection. We have identified the features of feedback that make it more effective as a trigger for reflection, notably, emotional salience and personal relevance. We found, however, that staff did not always recognize and respond to prompts for reflection that arose from patient feedback, either because the prompt was not acknowledged as a stimulus for reflection or because they lacked the capacity or opportunity to actively engage in reflection in the context of their clinical practice. Apart from appraisals, revalidation and responding to complaints—all mandatory and described by some as ‘ritualistic’,^22^ there were few occasions where staff mentioned being actively encouraged to reflect on patient experience data, and few opportunities in routine clinical practice for staff to take time to reflect. We did not focus in this paper on describing reflective activities or exploring the broader barriers and facilitators to reflection in practice, such as organizational resources or infrastructure, but this will be the focus of a subsequent paper.

Although trusts have well‐established systems for using patient feedback, particularly negative feedback, for quality improvement, there is a lack of infrastructure to enable improvement through reflection in practice. We need to consider how to provide the tools and create an environment that supports reflection in practice, enabling attitude and behaviour change. Deeper cognitive processing is dependent on ability to process, including capacity to engage with the message.^36^ As such, effective reflection is dependent on staff having the ability to process—for example there might have been an effective trigger but the ability to reflect may be limited through stress, overwork, tiredness and burnout; in addition, negative feedback can be demoralizing. Work is needed to understand how staff can be supported to enable them to have capacity to reflect, as well as having opportunities to engage in reflection in their day‐to‐day clinical practice. While toolkits have been developed to support the use of patient experience feedback for quality improvement,^29^, ^37^ no equivalent toolkit exists for the use of feedback in reflection. As part of the wider Pearl study, we aim to map barriers and enablers to embedding reflection in clinical practice based on behaviour change theory 38 and to develop a practical toolkit to support reflection on the patient experience in practice.

Our research involved in‐depth study of the use of patient experience feedback for reflection, and reflection in practice, across three trusts, including eight individual acute care units. A wide range of staff were interviewed and observed within the acute care settings so that the views of medical, nursing, administrative and managerial staff were captured. The study only encompassed three sites and focussed on acute care settings; while this might limit generalizability, the findings resonate with other studies investigating patient experience which have taken place in other health‐care environments.^29^, ^39^ Staff who agreed to be interviewed may be biased towards the importance of patient experience and reflective practice, and however, dissenting views were heard during the interviews and casual conversations. We conducted the research in two types of acute care unit, AMUs and ICUs. This enabled us to gain insight into reflection in practice across a range of settings. We have focused in this paper on commonalities in staff response to patient feedback across these settings. We did not attempt to make comparisons across the different types of units, although we acknowledge that the nature of patient feedback in each unit was qualitatively different—in particular, because patients tended to have longer stays on ICUs staff had more opportunity to get bedside feedback from patients and relatives, were more likely to receive cards and chocolates, and to hear from patients who returned to the unit following discharge. Taking into account, these local contextual differences will be important in efforts to develop interventions to support reflection in practice.

## 5 CONCLUSION

Most formal organizational‐level feedback of patient experience lacks immediacy for many staff and therefore tends not to stimulate reflective learning. The free‐text responses from surveys and hearing the patient stories at coffee mornings tend to have more impact on staff than aggregated quantitative data. Individuals are prompted to reflect when receiving informal personal feedback from patients, relatives or other members of staff, but this feedback is largely unrecognized at an organizational level. Staff value positive feedback, while organizations tend to respond to negative feedback such as complaints. All types of patient experience feedback– formal and informal, qualitative and quantitative, positive and negative–have the potential to stimulate reflective learning for staff in acute care settings, but maximizing this potential requires work to support staff in recognizing triggers for reflection and having the capacity and opportunity to reflect and learn from patient experience feedback.

## CONFLICT OF INTEREST

The authors declare that there is no conflict of interest.

## Data Availability

The data that support the findings of this study are available from the corresponding author upon reasonable request.
